# Psycho-behavioural responses of the general population to COVID-19 after mass vaccination: a cross-sectional study

**DOI:** 10.1017/gmh.2022.45

**Published:** 2022-08-02

**Authors:** Wai Tong Chien, Alice Wai Yi Leung, Stanley Kam Ki Lam, Kai Chow Choi, Cho Lee Wong

**Affiliations:** Faculty of Medicine, The Nethersole School of Nursing, The Chinese University of Hong Kong, Hong Kong SAR, China

**Keywords:** Anxiety, coronavirus, COVID-19, depression, general population, precautionary measures, psycho-behavioural responses, stress

## Abstract

**Background:**

Previous studies have examined public psycho-behavioural responses in the early stages of the epidemic, little is known after mass vaccination has been implemented. This study aimed to investigate the public's behavioural (adoption of COVID-19 precautionary measures) and psychological (depression, anxiety and stress) responses to COVID-19 and their relationships after the launch of the territory-wide vaccination programme in Hong Kong.

**Methods:**

A cross-sectional survey study using anonymous online or face-to-face questionnaires was conducted between June 2021 and September 2021. A convenience sample of Hong Kong Chinese residents aged ⩾18 years were recruited online by referrals and from a university-run community vaccination centre.

**Results:**

A total of 1893 valid questionnaires were received. The results showed that Hong Kong residents have high levels of adoption of precautionary measures and low levels of depression, anxiety and stress after the mass vaccination. Hierarchical regression analysis identified that in the fully adjusted model, the adoption of precautionary measures was a consistent protective factor (*β* ranged −1.51 to −1.67, *p* < 0.001) for depression, anxiety and stress amid the COVID-19 pandemic.

**Conclusions:**

This study offers new information on the public's psycho-behavioural responses to the pandemic, as well as insights into public health planning after introducing the mass vaccination.

## Introduction

The coronavirus disease 2019 (COVID-19) pandemic has infected and killed millions of people around the world (Coronavirus Resource Center Johns Hopkins University, 2022). Beyond being a global health crisis, the pandemic has disrupted every aspect of life such as learning, working and socialising. The early virus containment strategies mainly revolved around social distancing (such as quarantines, travel restrictions and the closure of premises and public places) and personal hygiene practices (such as hand hygiene and mask-wearing). High adherence to the precautionary measures is successful in mitigating the transmission; however, the unintended social, economic and health consequences of social distancing could impose detrimental effects on the psychological wellbeing of diverse populations (Douglas *et al*., 2020).

A growing body of evidence reveals the negative psychological responses of worldwide populations to the COVID-19 pandemic (Chow *et al*., 2020; Vindegaard and Benros, 2020; Xiong *et al*., 2020; Necho *et al*., 2021; Wu *et al*., 2021). For instance, in the early stages of the COVID-19 pandemic, the prevalence(s) of psychological distress including anxiety, depression and stress in the general populations of eight countries, including China, Spain, Italy, Iran, Turkey, Nepal, Denmark and the USA, were estimated to be 6–51, 15–48 and 8–82%, accordingly (Xiong *et al*., 2020). A later multinational study of 8559 community-dwelling adults from 17 countries in Asia Pacific and Middle East regions estimated that 69% of the participants experienced moderate to very high levels of psychological distress. The prevalence of the levels of distress varied by countries of residence, ranging from 45.9% in Thailand to 90.6% in Egypt (Rahman *et al*., 2021). In Hong Kong, the temporal trends of probable anxiety and depression throughout 2020 were estimated to be around 10–20%; notably, peaks were recorded during high infection rate periods or when social distancing measures were prolonged (Liao *et al*., 2021).

Simultaneously, a growing body of research focuses on the relationships between psychological responses to the pandemic and adoption of precautionary measures, being the common behavioural responses to the pandemic. Nevertheless, the direction of such relationship has been inconsistent; and the types of behavioural responses varied across studies. In line with the presumption that social distancing can be detrimental to psychological wellbeing, a majority of recent studies investigating social distancing behaviours reported that greater adoption of such measures was associated with higher levels of psychological distress (Benke *et al*., 2020; Marroquín *et al*., 2020; Kwok *et al*., 2021). By contrast, Zhao *et al*. (2020) found that engaging in social distancing behaviours might strengthen the feelings of security, which could help alleviate people's negative psychological responses to the pandemic (Zhao *et al*., 2020). Precautionary measures are not limited to social distancing measures; other measures such as mask-wearing and hygiene practices could be equally important. In view of the scant and inconclusive research on the relationships between psychological and behavioural responses to the pandemic (Padmanabhanunni and Pretorius, 2021; Wong and Alias, 2021), further study is warranted to ascertain such important relationships.

With the advent of the COVID-19 vaccine, many countries or regions launched free mass vaccination programmes (Mathieu *et al*., 2021). As of 8 February 2022, more than 4 billion people have been fully vaccinated, accounting for 53% of the global population (Coronavirus Resource Center, 2022). Hong Kong has been implementing the territory-wide COVID-19 vaccination programme since late February 2021, providing all Hong Kong residents with free vaccination services. Eligible residents can choose to receive CoronaVac (Sinovac) inactivated vaccine (for people 18 years of age or above) or Comirnaty (BioNTech) mRNA vaccine (for people 12 years of age or above). As of 8 February 2022, more than 4.8 million Hong Kong residents have been fully vaccinated, accounting for 72.5% of the total population over aged 12 years (The Government of Hong Kong Special Administrative Region, 2022). Given the emergence of more deadly and contagious variants of COVID-19 and the recent reports of breakthrough infection due to a decrease of the level of antibodies after vaccination, substantial efforts have been made to increase the vaccination rate, and/or introduce a second booster (Tré-Hardy *et al*., 2021; news.gov.hk, 2022).

To date, research on the psycho-behavioural responses to COVID-19 was largely based on studies conducted prior to the launch of mass vaccination. Despite sparks of hope through increased vaccination coverage, the impact of vaccination on psycho-behavioural responses to the COVID pandemic is largely unknown (Chong *et al*., 2021; Wong *et al*., 2021). Uncertainty remains as to whether vaccination uptake will impact the public's vigilance about infection control and/or related public health measures, as well as their psychological distress. Regular monitoring of psycho-behavioural responses during an infectious disease outbreak is critical as it can help identify behavioural gaps, inform infection control strategies to control/limit virus spread and inform policy efforts to mitigate the psychological impact of the pandemic (Wong and Alias, 2021). Therefore, this study aimed to investigate the psychological (depression, anxiety and stress) and behavioural (adoption of COVID-19 precautionary measures) responses of the Hong Kong general population following the launch of a territory-side vaccination programme, and their relationships during this unique and important period.

## Materials and methods

### Study design

We conducted a cross-sectional survey study using anonymous online or face-to-face questionnaires to collect data on the psycho-behavioural responses of Hong Kong residents to the COVID-19 pandemic between June 2021 and September 2021. During this period, only sporadic local cases of COVID-19 were recorded.

### Participants

To facilitate the inclusion of people with different levels of digital literacy in the community, a convenience sample of Hong Kong general population were recruited online and from one district Community Vaccination Centre under the COVID-19 Vaccination Programme (The Government of Hong Kong Special Administrative Region, 2022). To be eligible to participate in this study, community members were (1) aged 18 years or above; (2) able to understand written Chinese (online) or communicate in Cantonese/Mandarin (face-to-face). The sample size was estimated to ensure adequate study power to detect associations between psychological responses and behavioural responses to the COVID-19 pandemic. Using the power analysis software PASS 16.0 (NCSS, Kaysville, USA), we estimated that a sample size of 780 participants could detect an association with an effect size of *R*^2^ as small as 1% (i.e. behavioural responses explained at least 1% of variability of their psychological responses to the pandemic) with 80% power at 5% level of significance, by using linear regression. We originally planned to recruit the required number of participants (*N* = 780) into the study over a 4-month period, but the actual subject recruitment was much better than the minimum sample size required. We finally recruited 1960 participants over 4 months.

### Measures

The survey consisted of three sections: assessing behavioural responses to COVID-19, psychological responses to COVID-19 and participants' socio-demographic and background information. The survey took about 10 min to complete.

Behavioural responses to COVID-19 were assessed by a 10-item questionnaire developed by our research team in a similar recent study (Wong *et al*., 2020). The questionnaire covered precautionary measures such as personal hygiene practices and maintaining social distance. Each item was rated on a four-point Likert scale ranging from 1 (never) to 4 (always). Mean total score of the 10 items was calculated to indicate the respondents' behavioural responses. A higher total score indicated a higher degree of adoption of precautionary measures. Cronbach's *α* of the scale in the earlier study (Wong *et al*., 2020) and this study was 0.85 and 0.79, respectively.

Psychological responses in terms of depression, anxiety and stress were assessed using the 21-item Depression, Anxiety, and Stress Scale (DASS-21) (Lovibond and Lovibond, 1995). The scale was composed of three seven-item domains (depression, anxiety and stress) rated on a four-point Likert scale from 0 (does not apply to me at all) to 3 (applies to me very much or most of the time). The items scored on each domain were summed and then multiplied by 2 to provide the final domain score. A higher domain score indicated a higher level of depression, anxiety or stress (Lovibond and Lovibond, 1995). The three domain scores indicated the severities of three aspects of psychological distress accordingly with reference to the published norms (Lovibond and Lovibond, 1995). The normed levels of severity were: (i) depression: normal (0–9), mild to moderate (10–20) and severe to extremely severe (21–42); (ii) anxiety: normal (0–7), mild to moderate (8–14) and severe to extremely severe (15–42); and stress: normal (0–14), mild to moderate (15–25) and severe to extremely severe (26–42). The Chinese/English version of DASS-21 has been frequently used to assess the psychological responses of the general population in Hong Kong and middle-income Asian countries during the initial phase of the COVID-19 pandemic (Tso and Park, 2020; Wang *et al*., 2021). The Chinese version of DASS-21 has demonstrated high internal consistency (Cronbach's *α* > 0.8) and cross-cultural validity in Chinese clinical and non-clinical samples (Wang *et al*., 2016). Cronbach's *α*s of the depression, anxiety and stress domains of the Chinse version of DASS-21 in this study were 0.88, 0.84 and 0.88, accordingly.

Participants' socio-demographic and background information consisted of three sub-sections, including (i) socio-demographic characteristics (age, gender, place of birth, living status, marital status, highest educational qualification and current employment condition); (ii) health condition and lifestyle characteristics (mainly co-morbidities, smoking and alcohol drinking status and perceived physical health status); and (iii) experience or perceptions related to COVID-19 (financial impact, contact with known/suspected COVID-19 cases, perceived knowledge about COVID-19 and its vaccines, status of COVID-19 vaccination and perceived risk of COVID-19). All of the above information (except perceived risk of COVID-19) was assessed by a single-item question. The perceived risk of COVID-19 was assessed using a seven-item questionnaire developed by our research team in another recent study (Wong *et al*., 2020). Each item was scored on a five-point Likert scale from 1 (strongly disagree) to 5 (strongly agree). Mean total score of the seven items was calculated to indicate the respondents' risk perception. A higher score indicated a higher level of risk perception. Cronbach's *α* coefficient of 0.73 indicated satisfactory internal consistency.

### Data collection

Data were collected either through an online survey portal (in SurveyMonkey) created by our research team, or a face-to-face administered survey at a university-run Community Vaccination Centre under study. For the online survey, an invitation hyperlink was sent to staff of a local university, and they were asked to distribute the link to their networks (e.g. students, friends and relatives). Interested parties who clicked the invitation hyperlink were directed to the study information page in the online survey portal, followed by a request for consent (on the start page). After clicking the ‘Yes’ button to indicate their consent to the study, they were directed to the survey questionnaire online. For face-to-face survey, people who received the first or second dose of COVID-19 vaccination at the Community Vaccination Centre were invited to complete the paper version of the survey on site. A research staff approached eligible participants and explained the details of the study and their rights by referring to the hard copy of the study information sheet. After providing their written consent, participants completed the self-administered survey questionnaires and returned them to the research staff before leaving the centre.

### Statistical analysis

Normality of variables with continuous data was assessed using skewness statistic and normal probability plot; none of them deviated much from normal distribution. Participants' characteristics, including socio-demographics, health condition and lifestyle characteristics, experience and perception towards COVID-19, behavioural responses to COVID-19, and depression, anxiety and stress, were presented using frequency (percentage) and mean (standard deviation), as appropriate.

Linear regression analyses were performed to examine the associations of depression, anxiety and stress with behavioural responses to COVID-19. Specifically, a hierarchical approach was used for the regression analyses with the following batches of covariates successively inserted into the adjusted models: (1) socio-demographic characteristics; (2) health condition and lifestyle characteristics; and (3) experience or perception related to COVID-19. All statistical analyses were performed using IBM SPSS 26.0 (IBM Crop, Armonk, NY, USA). All statistical tests were two-sided with the level of significance set at 0.05.

### Ethical considerations

Ethical approval was obtained from the Survey and Behavioural Research Committee of The Chinese University of Hong Kong (SBRE-20-784). This study was conducted in compliance with the Declaration of Helsinki. Participants were informed of their rights to withdraw, their confidentiality and details of the study through online/written informed consent prior to data collection.

## Results

### Participants' characteristics

A total of 384 attempted the online survey, and 1576 community members completed the survey questionnaires at the Community Vaccination Centre. Owing to high levels of missing data in some questionnaires (>30% incompletions of the items), 67 participants (i.e. 26 in the online survey and 41 participants in the community vaccination centre) were excluded from the analysis. Therefore, the final sample for data analysis was 1893 participants. Table 1 summarises the characteristics of the participants. Most of them were aged 30–59 years (60.6%), female (61.3%) and reported having a source of income (72.2%). Most of the participants were born in Hong Kong (86.4%) and living with family members (92.6%), did not have any chronic medical disease (85.4%) and did not smoke (88.5%). Around half of them were cohabitating/married (49.5%), had a bachelor's degree or above (54.6%), had not consumed alcohol in the past 4 weeks (49.0%) and reported good/excellent physical state (44.9%).

**Table 1.** Characteristics of the study sample

	*N*	*n* (%)/mean (standard deviation)
*Socio-demographic characteristics*		
Age (years)	1852	
<18		91 (4.9%)
18–29		474 (25.6%)
30–59		1122 (60.6%)
⩾60		165 (8.9%)
Gender	1887	
Female		1156 (61.3%)
Male		731 (38.7%)
Birth in Hong Kong	1887	
No		257 (13.6%)
Yes		1630 (86.4%)
Living status	1887	
Live without family members		139 (7.4%)
Live with family members		1748 (92.6%)
Marital status	1878	
Single/divorced/widowed		948 (50.5%)
Cohabiting/married		930 (49.5%)
Highest educational qualification	1887	
Secondary/higher secondary/grade 7 to 12 or below		578 (30.6%)
Certificate/diploma/trade qualifications		278 (14.7%)
Bachelor/masters/PhD		1031 (54.6%)
Current employment condition	1864	
Unemployed/home maker (no source of income)		425 (22.8%)
Jobs affected by COVID-19		94 (5.0%)
Have an income source		1345 (72.2%)
*Health conditions and lifestyle characteristics*		
Chronic medical conditions	1845	
No		1576 (85.4%)
Yes		269 (14.6%)
Perceived physical health status	1846	
Poor/fair		105 (5.7%)
Average		912 (49.4%)
Good/excellent		829 (44.9%)
Smoking status	1847	
Never smoker		1634 (88.5%)
Ever smoker		213 (11.5%)
Current alcohol drinking (in the last 4 weeks)	1846	
No		905 (49.0%)
Yes		941 (51.0%)
*Experience or perceptions related to COVID-19*		
COVID-19 impacted financial situation	1878	
No impact		1324 (70.5%)
Yes, impacted positively		125 (6.7%)
Yes, impacted negatively		429 (22.8%)
Contact with known/suspected case of COVID-19	1841	
No		1640 (89.1%)
Yes		51 (2.8%)
Unsure		150 (8.1%)
Perceived knowledge about COVID-19	1875	
Poor/fair		70 (3.7%)
Average		1255 (66.9%)
Good/excellent		550 (29.3%)
Perceived knowledge on COVID-19 vaccine	1877	
Poor/fair		105 (5.6%)
Average		1273 (67.8%)
Good/excellent		499 (26.6%)
Status of COVID-19 vaccination	1866	
Haven't yet vaccinated		140 (7.5%)
Received one dose		377 (20.2%)
Received two doses		1349 (72.3%)
Perceived risk of COVID-19	1872	3.1 (0.6)^#^

Data of variables marked with # are presented as mean (standard deviation), all others are as frequency (%).

Regarding the experience and perception regarding COVID-19, the majority of participants perceived that their financial situations were not affected by the pandemic (70.5%), and had an average level of knowledge about COVID-19 (66.9%) and/or COVID-19 vaccines (67.8%). Only 2.8% reported known/suspected contact to COVID-19 cases. Notably, 72.3% of participants received two doses of COVID-19 vaccines, whereas only 7.5% did not receive any COVID-19 vaccine. The mean perceived COVID-19 risk score was 3.1 (s.d. = 0.6) out of 5.0 as maximum. The majority of participants agreed/strongly agreed that COVID-19 was a serious disease (73.2%) and feared that they would be infected (64.8%). If they were infected with COVID-19, most of them indicated that their health would be seriously affected (70.2%). However, less than 10% perceived that they (8.5%) and/or their family members (9.8%) were at risk of COVID-19 infection.

### Behavioural responses to COVID-19

The mean behavioural response score was 3.2 (s.d. = 0.5) out of 4.0 as maximum (Table 2). The most frequent precautionary measure taken by participants was to wear a surgical mask (84.9%) when taking public transport or staying in a crowded venue, followed by putting down the toilet lid before flushing (67.4%) and avoiding non-essential travel outside Hong Kong (64.0%). By contrast, only 10.6% of participants always followed the health authority's advice of ‘Go out less and reduce social activities and maintain an appropriate social distance with others’.

**Table 2.** Behavioural responses to COVID-19

	*N*	Never *n* (%)	Sometimes *n* (%)	Often *n* (%)	Always *n* (%)	Mean (s.d.)^a^
1. Go out less and reduce social activities and maintain an appropriate social distance with others.	1893	56 (3.0)	831 (43.9)	805 (42.5)	201 (10.6)	2.6 (0.7)
2. Avoid non-essential travel outside Hong Kong.	1892	80 (4.2)	178 (9.4)	423 (22.4)	1211 (64.0)	3.5 (0.8)
3. Avoid touching animals, poultry/birds or their droppings.	1892	213 (11.3)	293 (15.5)	538 (28.4)	848 (44.8)	3.1 (1.0)
4. Wear a surgical mask when taking public transport or staying in a crowded place.	1893	7 (0.4)	39 (2.1)	239 (12.6)	1608 (84.9)	3.8 (0.5)
5. Perform hand hygiene before wearing and after removing a mask	1893	19 (1.0)	221 (11.7)	609 (32.2)	1044 (55.2)	3.4 (0.7)
6. Wash hands with liquid soap and water and rub for at least 20 s.	1892	31 (1.6)	333 (17.6)	735 (38.8)	793 (41.9)	3.2 (0.8)
7. Perform hand hygiene with 70–80% alcohol-based handrub if hand washing facilities are not available.	1892	17 (0.9)	239 (12.6)	639 (33.8)	997 (52.7)	3.4 (0.7)
8. Put down the toilet lid before flushing	1893	21 (1.1)	153 (8.1)	444 (23.5)	1275 (67.4)	3.6 (0.7)
9. Pour half a litre of water into each drain outlet every week.	1893	264 (13.9)	666 (35.2)	543 (28.7)	420 (22.2)	2.6 (1.0)
10. Follow the updates about the spread of the virus.	1891	18 (1.0)	384 (20.3)	828 (43.8)	661 (35.0)	3.1 (0.8)
Overall mean score	1888					3.2 (0.5)

a Scale ranged from 1 to 4. 1: never; 2: sometimes; 3: often; 4: always.

### Psychological responses to COVID-19: depression, anxiety and stress

The mean scores of the DASS-21 subscales on depression, anxiety and stress were 5.6 (s.d. = 7.2), 5.5 (s.d. = 6.3) and 8.3 (s.d. = 8.0), accordingly. According to the cut-off scores proposed by Lovibond and Lovibond (Lovibond and Lovibond, 1995), less than or about one-fifth of participants reported mild to moderate levels of depression (18.3%), anxiety (20.2%) and stress (14.2%); and less than one-tenth experienced severe to extremely severe levels of depression (4.8%,), anxiety (8.5%) and stress (4.4%).

### Associations of depression, anxiety and stress with behavioural responses to COVID-19

We conducted hierarchical regression analyses using depression, anxiety or stress as dependent variables, behavioural responses as independent variables in model 1, further controlling for socio-demographic characteristics in model 2, health conditions and lifestyle characteristics in model 3, as well as experience or perceptions related to COVID-19 in model 4 (Table 3). The results suggested that the mean behavioural responses score remained negatively significantly associated with depression, anxiety and stress in model 1 (unstandardised regression coefficient, *β* = −1.58 to −2.13, *p* < 0.001), model 2 (*β* = −1.71 to −2.01, *p* < 0.001), model 3 (*β* = −1.39 to −1.74, *p* < 0.001) and model 4 (*β* = −1.51 to −1.67), accordingly. The results indicate that behavioural responses to COVID-19 were a consistent protective factor for depression, anxiety and stress independently.

**Table 3.** Association between behavioural responses against COVID-19 and psychological responses assessed by DASS-21

	Depression subscale		Anxiety subscale		Stress subscale	
	*β* (95% CI)	*p* value	*β* (95% CI)	*p* value	*β* (95% CI)	*p* value
Model 1						
Behavioural responses against COVID-19	−2.13 (−2.87 to −1.40)	<0.001	−1.83 (−2.47 to −1.19)	<0.001	−1.58 (−2.41 to −0.75)	<0.001
*R*^2^	0.019		0.018		0.008	
Model 2						
Behavioural responses against COVID-19	−2.01 (−2.77 to −1.26)	<0.001	−1.84 (−2.50 to −1.17)	<0.001	−1.71 (−2.56 to −0.86)	<0.001
*R*^2^	0.047		0.045		0.042	
*R*^2^ change	0.029	<0.001	0.027	<0.001	0.034	<0.001
Model 3						
Behavioural responses against COVID-19	−1.74 (−2.48 to −1.01)	<0.001	−1.60 (−2.24 to −0.96)	<0.001	−1.39 (−2.21 to −0.57)	<0.001
*R*^2^	0.116		0.120		0.121	
*R*^2^ change	0.068	<0.001	0.075	<0.001	0.078	<0.001
Model 4						
Behavioural responses against COVID-19	−1.67 (−2.40 to −0.93)	<0.001	−1.65 (−2.30 to −1.00)	<0.001	−1.51 (−2.34 to −0.69)	<0.001
*R*^2^	0.138		0.138		0.146	
*R*^2^ change	0.023	<0.001	0.018	<0.001	0.025	<0.001

*β*, unstandardised regression coefficient; CI, confidence interval.

Model 1 (unadjusted model): with only behavioural responses to COVID-19 included.

Model 2: with adjustment for socio-demographic characteristics, including age, gender, birth in Hong Kong, living status, marital status, highest education qualification, current employment condition.

Model 3: with adjustment for the covariates in model 2 + health conditions and lifestyle characteristics, including chronic medical condition, perceived physical health status, smoking status, alcohol drink.

Model 4: with adjustment for the covariates in model 3 + experience or perception related to COVID-19, including perceived risk of COVID-19, impact of COVID-19 on financial situation, contact with known/suspected cases of COVID-19, experience related to COVID-19 pandemic, perceived knowledge on COVID-19, status of COVID-19 vaccination.

## Discussion

This was one of the first few studies to provide evidence of psycho-behavioural responses of the general population to the COVID-19 pandemic following mass vaccination in Hong Kong and worldwide. During the data collection, Hong Kong was experiencing the fourth wave of COVID-19 outbreak and had launched the vaccination programme for about 3 months; the changes brought about by the COVID-19 pandemic and subsequent measures taken to limit the spread of the virus had substantially affected the lives of the public and individuals. This situation offered an ideal opportunity to examine the behavioural responses of the general population and repercussions on the psychological responses to COVID-19 during this unique period. The results reveal that most participants were compliant to most of the recommended precautionary measures, and only a small proportion of participants experienced moderate to high levels of depression, anxiety and/or stress.

Before mass vaccination, Chinese have demonstrated a higher adoption of precautionary measures (such as face mask use) than Europeans, which is probably attributed to the influence of collectivism in Chinese culture and individualism in European culture (Wang *et al*., 2020*a*). This study demonstrated that Hong Kong people had adopted or continue to take most precautionary measures after mass vaccination, including wearing surgical masks and avoiding non-essential travel. However, the precautionary measures had gaps. For instance, only half of participants continued to comply with hand hygiene recommendations; an even lower percentage of them continued to practise social distancing (11%). In fact, a high level of compliance with social distancing was reported during the first three waves of COVID-19 outbreaks when vaccines were not yet available (Wong *et al*., 2020; Kwok *et al*., 2021). The low compliance reported in this study indicated a decline in adherence following the launch of the vaccination programme. This change may also be partly attributable to the gradual relaxation of social distancing measures under the ‘vaccine bubble’ scheme (The Government of Hong Kong Special Administrative Region, 2021). For example, restaurants could extend business hours and increase the seating capacity from four to 12 per table if at least two-thirds of customers have received the first dose. Notwithstanding, the gaps in compliance with certain precautionary measures identified in this study deserve attention. Given that social distancing remains an important strategy to curb the resurgence of COVID-19 and requires long-term collective effort (Center for Health Protection, 2021; Huang *et al*., 2021; Sills *et al*., 2021), the results call for effective health communication that emphasises the importance of avoiding unnecessary gatherings and implementing precautionary measures continuously in an effort to minimise the transmission of COVID-19. For example, existing health promotion programmes should be refined to highlight the importance of adhering to these precautionary measures following mass vaccination.

Compared with similar studies using DASS-21, the mean scores of depression, anxiety and stress were significantly lower than those reported by similar cohorts during the second wave of COVID-19 outbreak in Hong Kong (Tso and Park, 2020), and slightly lower than those of the general population in mainland China in early 2020 (Wang *et al*., 2021). These findings imply that the impact of COVID-19 on psychological difficulties would gradually diminish as people adjust to the new normal. However, this improvement may be partly due to the government's promotional initiatives on mental health in response to the COVID-19 pandemic since the first wave of outbreak (The Government of Hong Kong Special Administrative Region, 2020). Furthermore, this improvement might be attributed to the protective effect of COVID-19 vaccination on mental health (Hao *et al*., 2021; Chen *et al*., 2022). Nevertheless, long-term psychological responses fluctuate with disease progression, availability of effective treatments, public health initiatives (Gloster *et al*., 2021) and timely dissemination of up-to-date and accurate COVID-19 health information (Wang *et al*., 2020*b*). Further national and international investigation of the general public's and/or high-risk groups' psychological responses in the trajectory of the pandemic is warranted.

This study showed that behavioural responses to COVID-19 (adoption of various types of precautionary measures) were a salient determinant of depression, anxiety and stress in the general population. Specifically, greater adoption of precautionary measures was a common protective factor for depression, anxiety and stress following mass vaccination implementation. In fact, the relationship between behavioural and psychological responses to the pandemic has been studied prior to mass vaccination with mixed results yielded (Benke *et al*., 2020; Marroquín *et al*., 2020; Zhao *et al*., 2020; Kwok *et al*., 2021; Padmanabhanunni and Pretorius, 2021; Wong and Alias, 2021). However, during periods of high infection rates and strict social distancing measures, adoption of social distancing measures was positively associated with anxiety, depression and stress (Benke *et al*., 2020; Marroquín *et al*., 2020; Kwok *et al*., 2021). This study was conducted during a period of low infection rates and relaxed social distancing measures. In this context, the negative psychological impacts associated with social distancing may be reduced. Moreover, engaging in precautionary measures may promote altruistic emotions (feeling good about their contribution to community health), thereby improving psychological wellbeing (Post, 2005). Further empirical research is needed to confirm this proposition.

Taken together, our results shed light on policy enactment for optimising mental health promotion efforts by encouraging long-term maintenance of precautionary measures against COVID-19 at an individual level. Considering the high vaccination rate (72.3%) in Hong Kong, the results may be generalised to other countries with similar vaccination rates.

### Limitations

This study has a few limitations. Firstly, this cross-sectional study only collected data at a single time point; hence, the causal relationship between adoption of precautionary measures and psychological responses (including depression, anxiety and stress) could not be established. However, the findings offer a basis for testing a causal hypothesis. In addition, further study involving a longitudinal design is required to track temporal changes in psycho-behavioural responses to the COVID-19 pandemic. Secondly, compared with the latest census data (Census and Statistics Department, 2018), our sample was overrepresented by female participants (61.3%) and people with an educational attainment of a bachelor degree's or above (54.6%). Coupled with the use of convenience sampling strategies, in particular, the snowball sampling strategy for the online survey, the generalisability of the findings to the entire Hong Kong Chinese population was reduced. Thirdly, the depression, anxiety and stress outcomes were assessed using a self-reported scale and might not accurately reveal or be interpreted as objective clinical assessment or diagnostic data/findings. Finally, social desirability bias might exist in self-reported outcomes, which likely undermined the internal validity of the study findings.

## Conclusion

This study was one of the very few studies to investigate the psycho-behavioural responses of the general population to the COVID-19 pandemic after the implementation of mass vaccinations. Overall, participants were compliant to most of the recommended precautionary measures. However, gaps in precautionary measures were identified. Besides, a subgroup of participants experienced moderate to high levels of depression, anxiety and/or stress. The behavioural responses to COVID-19 were a salient determinant of depression, anxiety and stress among the general population. These findings provide new evidence and insights for successful control of COVID-19 infection and/or mitigating future infection outbreaks. Furthermore, the findings offer a better understanding of psycho-behavioural responses and their association to inform further efforts in mental health promotion and COVID-19 containment strategies in Hong Kong and other countries, which are on their way to herd immunity through vaccination.

## Data

The anonymous data which form the basis for this study are available from the authors on reasonable request.
